# Identification and Isolation Pattern of *Globisporangium* spp. from a *Sanionia* Moss Colony in Ny-Ålesund, Spitsbergen Is., Norway from 2006 to 2018

**DOI:** 10.3390/microorganisms9091912

**Published:** 2021-09-09

**Authors:** Motoaki Tojo, Natsumi Fujii, Hironori Yagi, Yuki Yamashita, Katsuyuki Tokura, Kenichi Kida, Akiho Hakoda, María-Luz Herrero, Tamotsu Hoshino, Masaki Uchida

**Affiliations:** 1 Laboratory of Plant Pathology, Graduate School of Life and Environmental Sciences, Osaka Prefecture University, Gakuen-Cho 1-1, Sakai, Osaka 599-8531, Japan; natu7230715@hotmail.com (N.F.); yagihironori.9@gmail.com (H.Y.); yukiyamashita@ao.osakafu-u.ac.jp (Y.Y.); k.tokura@asahi-kg.co.jp (K.T.); k-kida@kumiai-chem.co.jp (K.K.); chihiro1202@maia.eonet.ne.jp (A.H.); 2 Norwegian Institute of Bioeconomy Research (NIBIO), P.O. Box 115, NO-1431 Ås, Norway; maria.herrero@nibio.no; 3 Department of Life and Environmental Science, Faculty of Engineering, Hachinohe Institute of Technology 88-1, Obiraki, Myo, Hachinohe 031-8501, Japan; t-hoshino@hi-tech.ac.jp; 4 National Institute of Polar Research (NIPR), 10-3 Midori-cho, Tachikawa, Tokyo 190-8518, Japan; uchida@nipr.ac.jp

**Keywords:** Arctic region, long-term population changes, *Globisporangium*, *Pythium*, moss, plant pathogens

## Abstract

*Globisporangium* spp. are soil-inhabiting oomycetes distributed worldwide, including in polar regions. Some species of the genus are known as important plant pathogens. This study aimed to clarify the species construction of *Globisporangium* spp. and their long-term isolation pattern in *Sanionia* moss in Ny-Ålesund, Spitsbergen Is., Norway. *Globisporangium* spp. were isolated at two-year intervals between 2006 and 2018 at a *Sanionia* moss colony, Ny-Ålesund, Spitsbergen Is., Norway. The isolates were obtained by using three agar media and were identified based on sequences of the rDNA-ITS region and cultural characteristics. Most of the *Globisporangium* isolates obtained during the survey were identified into six species. All six species were grown at 0 °C on an agar plate and used to infect *Sanionia* moss at 4 and/or 10 °C under an in vitro inoculation test. The total isolation frequency of *Globisporangium* gradually decreased throughout the survey period. The isolation frequency varied among the six species, and four of the species that showed a high frequency in 2006 were rarely isolated after 2016. The results suggested that *Globisporangium* inhabiting *Sanionia* moss in Ny-Ålesund has a unique composition of species and that most of the species reduced their population over the recent decade.

## 1. Introduction

Plant pathogenic fungi and oomycetes can affect individual growth and community structure in many wild plants [1]. Warmer temperatures can increase the relative abundance of phytopathogenic fungi and oomycetes [2,3]. Plant pathogens can also occur on mosses and vascular plant species in the polar regions [4]. Svalbard, a High Arctic archipelago, has been investigated for plant pathogenic fungi and oomycetes since the late 19th century [5,6]. Many plant inhabitants have been found, including at least 173 species of fungi and 3 of oomycetes [7]. Fourteen fungal species were also recorded in soil of the archipelago [8]. Some of these fungi and oomycetes are thought to be plant pathogens or potential plant pathogens. The recent climate change is expected to have a significant impact on biological diversity in polar ecosystems [9]. For example, it is expected to lead to an increasing woody plant distribution range and to a decrease in the distribution of mosses and lichens [10]. Consequently, the diversity of mosses and lichens is expected to decrease [11,12]. On the other hand, the biomass of the shrub layer in the tundra is expected to increase [13]. Floristic variation may, in turn, affect microhabitat and microbial diversity. However, there have been very few reports regarding their distribution and pathogenic capacity in natural ecosystems in polar regions.

Mosses play an important role as primary producers of organic matter worldwide, including in polar regions [14]. *Sanionia uncinata* (Hedw.) is one of the dominant moss species in both the Arctic and Antarctic regions [15,16]. Brown discoloration of stem leaves has been commonly found on moss that inhabits wet ground where water accumulates after snow and ice have melted. It has been reported that *Sanionia* moss can be infected or actively attacked by ascomycete and basidiomycete fungi [17,18]. Microorganisms, especially fungi, act as decomposers of mosses and higher plants in polar regions [19]. Several studies have been published on fungi actively infecting mosses in the Arctic [18] and Antarctic [17,20,21]. However, there have been few reports of oomycetes as causal agents of damage to mosses [22].

*Globisporangium* is the major genus of the oomycete segregated from the genus *Pythium* [23,24]. *Globisporangium* spp. are cosmopolitan, and many species of this genus can infect a variety of host species [24]. Some species of the genus cause snow rot disease in winter wheat and barley [25]. *Globisporangium* has also been isolated from the brown discolored moss in the polar region [22]. It has also been identified as a potential plant pathogen on *Deschampsia antarctica* (Poaceae) in the maritime Antarctic [26]. Although these low-temperature *Globisporangium* spp. are less freeze-resistant than the low-temperature fungi [27], they can survive under freezing conditions by infecting living plant tissue [28]. However, there are few reports on their distribution and parasitism in natural polar ecosystems compared to those for fungi [4,22,29]. Also, *Globisporangium* spp. found in the *Sanionia* moss have not been properly identified and therefore are not in published records to date [22].

The objectives of this study were to clarify long-term population changes of *Globisporangium* spp. in the *Sanionia* moss in Ny-Ålesund, Spitsbergen Is., Norway, as well as confirm their species identities and infectivity to the moss.

## 2. Materials and Methods

### 2.1. Isolation

**Figure 1:**
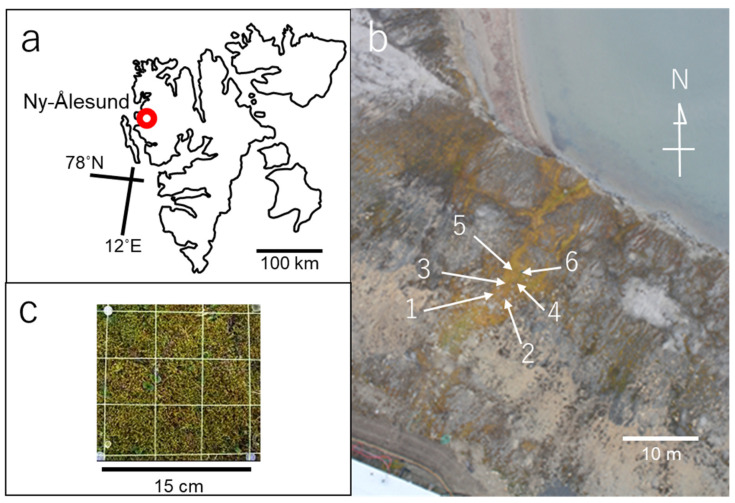
Study site and plot: (**a**) location of Ny-Ålesund, Spitsbergen Is., Svalbard Archipelago, Norway; (**b**) distribution of the six sampling plots (arrow heads) in the *Sanionia* moss colony at a north side cliff in Ny-Ålesund; (**c**) the 15 cm-square plot. The yellow threads of the quadrat were put on the moss surface only when the moss was sampled. The aerial photograph was kindly taken by Dr. Jun Inoue of the National Institute of Polar Research (NIPR).

Approximately 10 mm-long shoots of the *Sanionia* moss (*Sanionia uncinata* (Hedw.) Loeske) were sampled from six 15 cm-square plots in the moss colony at the north side cliff in Ny-Ålesund (78° 55′ 47″ N, 11° 56′ 08″ E, Figure 1), Spitsbergen Is., Norway in July to August in 2006, 2008, 2010, 2012, 2014, 2016. and 2018. The sample amount was limited to less than 4 g per plot to avoid damage to the moss colony. After washing in tap water and air drying, thirty-six shoots of the moss sample were placed on each of water agar (WA) and *Globisporangium* selective VP_3_ [30] and NARM [31] media. The moss shoots were incubated at 10 to 15 °C for one week on the media. Mycelia growing on the media were subcultured on corn meal agar (CMA; Becton Dickinson and Company, Franklin Lakes, NJ, USA) and maintained at 10 °C in the dark until use. Experiment was repeated for six 15 cm-square plots close to each other in the single moss colony (Figure 1b).

### 2.2. rDNA-ITS Analysis

All isolates obtained were compared with known species based on entire rDNA-ITS sequences. Genomic DNA of the obtained *Globisporangium* isolates was extracted from mycelium grown on V8 broth prepared according to Miller [32]. Mycelia were frozen in liquid nitrogen and ground using pestle and mortar. DNA extraction was performed using the DNeasy Plant kit (Qiagen, Hilden, Germany) following the manufacturer’s instructions, and the DNA was then stored at −20 °C until used.

Sequences of the ITS region containing ITS1 and ITS2 were determined as follows. In the polymerase chain reaction (PCR), primer pairs ITS5 (5′ GGAAGTAAAAGTCGTAACAAGG 3′) and ITS4 (5′ TCCTCCGCTTATTGATATGC 3′) described by White et al. [33] were used. Fifty microliters of PCR reaction mixture contained 25 µL 2×MightyAmp buffer ver. 2, 0.5 µM of each primer, 0.25 µL MightyAmp DNA polymerase (Takara Bio, Shiga, Japan), and 1 µL template DNA. Amplification was carried out in a PerkinElmer 9700 thermal cycler (PerkinElmer Inc., Waltham, MA, USA). The amplification program consisted of a predenaturation at 95 °C for 5 min; 35 cycles of 95 °C for 30 s, 55 °C for 30 s, and 72 °C for 1 min; and a final incubation at 72 °C for 7 min to complete the last extension. PCR products were used for sequence analysis.

The sequence reaction was performed using the primers ITS4 and ITS5. Products of the sequence reaction were analyzed with an ABI 3730 DNA Sequencer (Applied Biosystems). The sequences were aligned with relevant *Globisporangium* sequences obtained from the GenBank database using BLAST (http://www.ncbi.nlm.nih.gov/blast, accessed on 30 July 2021).

**Figure 2:**
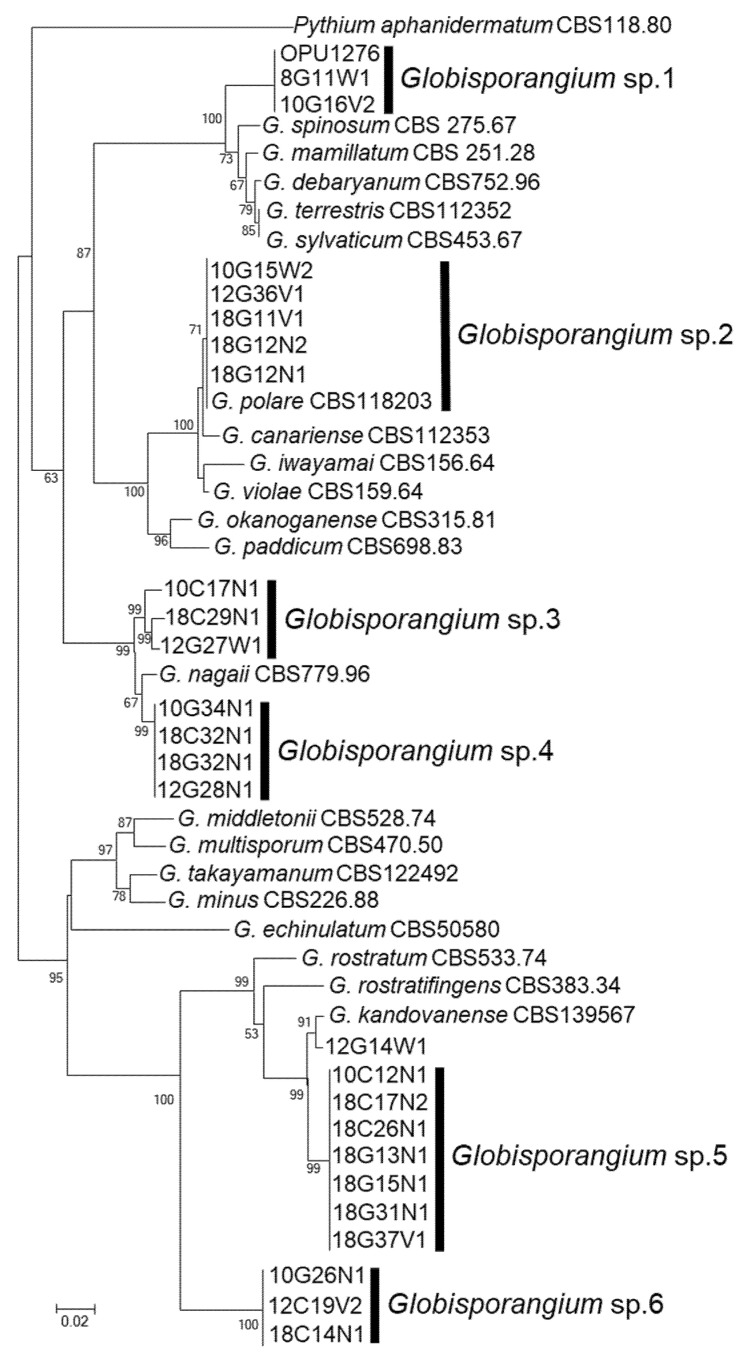
Phylogenetic positions of *Globisporangium* strains obtained from the *Sanionia* moss in Ny-Ålesund, Spitsbergen Island, Norway, on a neighbor-joining (NJ) tree of the ITS of the rDNA region. Numbers beside the branches are the bootstrap values (>50%) of 1000 replicates. *Pythium aphanidermatum* strain CBS118.80 was used as an outgroup. The six major subclades found in this study were named as *Globisporangium* spp. 1–6.

The BLAST search showed that the *Globisporangium* isolates obtained in this study were divided into six major taxonomic groups. A phylogenetic tree was therefore made based on randomly selected isolates from each of the major taxonomic groups (Figure 2, Table S1). The tree was constructed by MEGA version 5.2.2 [34] based on neighbor-joining (NJ) analysis [35]. To determine the support for each clade, a bootstrap analysis was performed with 1000 replications. *Pythium aphanidermatum* strain CBS118.80 was used as an outgroup.

### 2.3. Characterizations of Morphology and Hyphal Growth Speed

One *Globisporangium* strain from each of the six major taxonomic groups were used for characterization of morphology and hyphal growth speed. The strains used were 10G16V2, 10G15W, 10C17N1, 10G34N1, 10C12N1, and 10G26N1 (Figure 2, Table S1).

Morphology of the strains was examined in grass-leaf water culture [36]. All strains were grown on CMA, potato dextrose agar (PDA; Becton Dickinson and Company), or V8 juice agar at 4–17 °C. A piece of agar medium was placed in a Petri dish containing a shallow layer of sterilized water, to which some 1–2 cm leaf pieces of gramineous weeds sterilized by autoclave were added. After incubation at 4–17 °C until *Globisporangium* strains colonized the leaves, sterilized pond water was added. Spore formation and the shape of said spores were examined by optical microscope (Olympus BX 43, Tokyo, Japan).

To determine hyphal growth rates, the strains were incubated on potato carrot agar (PCA) prepared according to van der Plaats-Niterink [37] in Petri dishes at 0, 4, 7, 10, 13, 16, 19, 22, 25, 28, 31, 34, and 37 °C in darkness, and colony diameters were measured. The experiments were repeated three times with one plate per repetition.

### 2.4. Isolation Pattern

Isolation frequency was determined as the number of the moss shoots that isolated *Globisporangium* spp. divided by the total number of the moss shoots examined. The isolation frequency was compared yearly by least significant differences based on a Tukey–Kramer Honestly Significant Difference test (*p* < 0.05) by JMP 13 (SAS Institute, Cary, NC, USA).

### 2.5. Infectivity to Sanionia Moss

Eleven *Globisporangium* strains from the six major taxonomic groups were used to test infectivity to *Sanionia* moss (*S. uncinata*). The strains used were 10G16V2, 10G15W, 10C17N1, 10G34N1, 10C12N1, 10G26N1, 18G11V1, 18C29N1, 18C32N1, 18C17N2, and 18C14N1 (Figure 2, Table S1). Stem-leaf sections (15 mm long, 0.5 mm wide) of the *Sanionia* obtained in Ny-Ålesund were placed on plates containing a KNOP agar medium [38] amended with 1.5% agar and were grown in a growth chamber at 10 °C for 3–4 months with continuous light (80 mmol m^−2^ s^−1^ measured at the level of the plants). A CMA plug (8 mm diameter) from each strain of *Globisporangium*, grown at 15 °C for 1 week, was placed in the center of the plate containing the *Sanionia* moss sections. Uninfected CMA was used as a control. The plates were kept at 0 °C, 4 °C, and 15 °C in darkness for approximately one month in a growth chamber. Infectivity was confirmed by optical microscopic observation. Recovery of the inoculated *Globisporangium* strains from the infected stem leaves was done using NARM medium. There were 16 replicates for each strain, using one moss segment for each replicate.

## 3. Results and Discussion

### 3.1. Isolation and Identification

In total, 434 isolates of *Globisporangium* spp. were obtained from the *Sanionia* moss during the 2006–2018 survey. All the isolates obtained were compared with known species based on the entire rDNA-ITS sequences through the GenBank database. The phylogenetic analysis of the sequences revealed that all isolates obtained were divided into six taxonomic groups of *Globisporangium* spp., which formed each monophyletic clade based on neighbor-joining (NJ) analyses (Figure 2). There was one exception: strain 12G14W1 did not belong to any of the six taxonomic groups. Since the maximum identities of these taxonomic groups against known species [22,39,40,41] were low (86.7 to 96.7%, Table S1), with the exception of one group with 99.8–100% similarity to *G. polare* (Table S1), they were named as *Globisporangium* sp. 1, sp. 2 (=*G. polare*), sp. 3, sp. 4, sp. 5, and sp. 6 (Figure 2). *Globisporangium* strain 12G14W1 was isolated only once, in 2012. The phylogenetic position of the strain 12G14W1 was the closest to *G. kandovanense* [41], but further analysis was not conducted because of loss of the strain.

Characteristics of morphology and hyphal growth speed of *Globisporangium* spp. 1–6 are described below.

**Figure 3:**
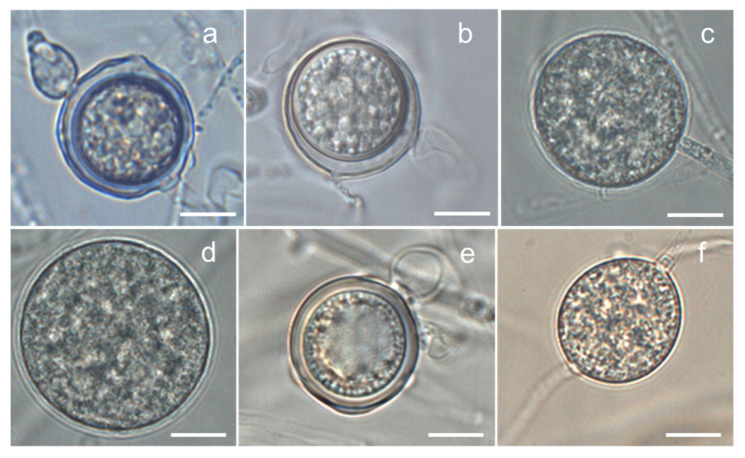
Oospores or sporangia of *Globisporangium* spp. isolated from the *Sanionia* moss in Ny-Ålesund, Spitsbergen Island, Norway: (**a**) aplerotic oospore of *Globisporangium* sp. 1 strain 10G16V2; (**b**) aplerotic oospore of *Globisporangium* sp. 2 strain 10G15W2 (=*G. polare*); (**c**) globose sporangium of *Globisporangium* sp. 3 strain 10C17N1; (**d**) globose sporangium of *Globisporangium* sp. 4 strain 10G34N1; (**e**) plerotic oospore of *Globisporangium* sp. 5 strain 10C12N1; and (**f**) hyphal swelling of *Globisporangium* sp. 6 strain 10G26N1. Bars = 10 μm.

**Figure 4:**
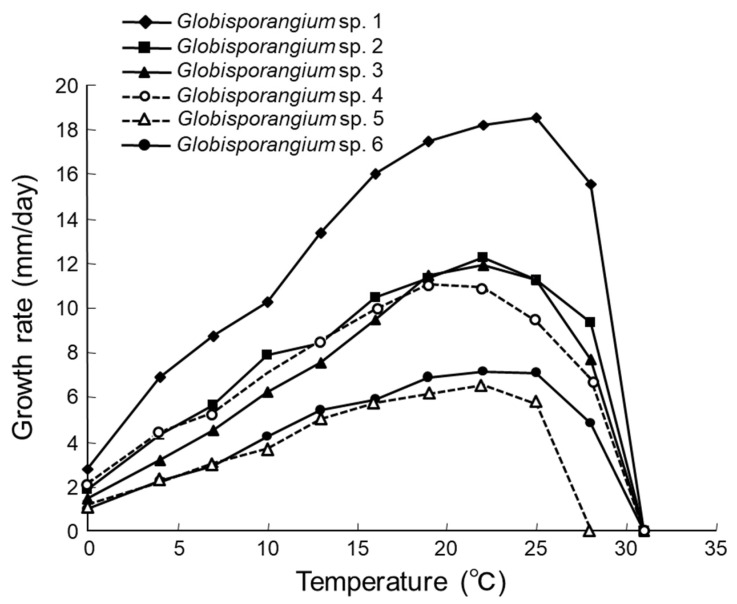
Mycelial growth rate of *Globisporangium* spp. isolated from a single colony of *Sanionia* moss in Ny-Ålesund, Spitsbergen Island, Norway on potato carrot agar at different temperatures in darkness. The strains used were *Globisporangium* sp. 1 strain 10G16V2, *Globisporangium* sp. 2 (=*G*. *polare*) strain 10G15W2, *Globisporangium* sp. 3 strain 10C17N1, *Globisporangium* sp. 4 strain 10G34N1, *Globisporangium* sp. 5 strain 10C12N1, and *Globisporangium* sp. 6 strain 10G26N1.

*Globisporangium* sp. 1 strain 10G16V2: Main hyphae were up to 5 µm in diameter. Sporangia were not observed. Hyphal swellings were observed in single culture. Oogonia did not develop in single culture, but developed in dual culture with OPU1276. Strain OPU1276 was isolated from the present study site in July 2003 and showed identical rDNA-ITS sequence with strain 10G16V2 (Figure 2, Table S1). Oogonia were globose (Figure 3a), smooth, terminal sometimes intercalary, and 20.0–24.5 (mean 21.7) µm in diameter. Antheridia were monoclinous, with 1–4 per oogonium. Oospores were aplerotic, globose, smooth, and 16.5–21.5 (mean 18.7) µm in diameter, with one per oogonium. The thickness of the oospore wall was 0.5–1.5 (mean 1.0) µm. The minimum, optimum, and maximum temperatures for growth on PCA were 0 °C, 25 °C, and 28 °C, with daily growth rates at 2.7 mm, 18.3 mm, and 15.7 mm, respectively (Figure 4). The strain did not grow at 31 °C but showed regrowth when the dishes were placed at 22 °C. *Globisporangium* sp. 1 was closely phylogenetically related to *G. spinosum, G. sylvaticum*, and *P. macrosporum* (Figure 2) but was distinguished from these known species by the size and shape of its oogonia.

*Globisporangium* sp. 2 strain 10G15W2: Main hyphae were up to 6 µm in diameter. Sporangia were terminal and globose or sometimes subglobose. Zoospores were formed at 4–15 °C. Oogonia did not develop in single culture but developed in dual culture with *G. polare* CBS118202 [22]. Oogonia were globose, smooth, terminal or sometimes intercalary, and 17.3–26.9 (mean 23.1) µm in diameter (Figure 3b). Antheridia were diclinous, with 1–3 per oogonium. Oospores were aplerotic, globose, smooth, and 14.4–24.5 (mean 19.7) µm in diameter, with one per oogonium. The thickness of the oospore wall was 0.7–1.5 (mean 1.0) µm. The minimum, optimum, and maximum temperatures for growth on PCA were 0 °C, 22 °C, and 28 °C, with daily growth rates at 1.7 mm, 12.1 mm, and 9.4 mm, respectively (Figure 4). The growth rate at 25 °C was 11.2 mm per day. Since these taxonomic features matched those of *G. polare*, *Globisporangium* sp. 2 was identified as *G. polare* [22]. The result of the morphological study is in concordance with the result of the phylogenetic study.

*Globisporangium* sp. 3 strain 10C17N1: Main hyphae were up to 5 µm in diameter. Globose sporangia were observed in single culture (Figure 3c). Sexual reproductive organs did not produce in single or dual culture. The minimum, optimum, and maximum temperatures for growth on PCA were 0 °C, 22 °C, and 28 °C, with daily growth rates at 1.1 mm, 11.9 mm, and 7.8 mm, respectively (Figure 4). The growth rate at 25 °C was 11.2 mm per day. *Globisporangium* sp. 3 did not grow at 31 °C but showed regrowth at 22 °C.

*Globisporangium* sp. 4 strain 10G34N1: Main hyphae were up to 5 µm in diameter. Globose sporangia were observed in single culture (Figure 3d). Sexual reproductive organs were not produced in single or dual culture. The minimum, optimum, and maximum temperatures for growth on PCA were 0 °C, 19 °C, and 28 °C, with daily growth rates at 2.1 mm, 11.0 mm, and 6.9 mm, respectively (Figure 4). The growth rate at 25 °C was 9.4 mm per day. The strain did not grow at 31 °C but showed regrowth at 22 °C.

*Globisporangium* spp. 3 and 4 were closely related to *G. nagaii* based on rDNA- ITS sequences (Figure 2). Since asexual stages of *Globisporangium* spp. 3 and 4 were not formed in this study, additional taxonomic study is needed to distinguish *Globisporangium* spp. 3 and 4 from *G. nagaii*.

*Globisporangium* sp. 5 strain 10C12N1: Main hyphae were up to 6 µm in diameter. Globose sporangia, hyphal swellings, and sexual reproductive organs were observed in single culture. Oogonia were globose, smooth, terminal, and 20.0–26.0 (mean 22.9) µm in diameter (Figure 3e). Antheridia were monoclinous or occasionally diclinous, with 1–2 per oogonium. Oospores were aplerotic or occasionally plerotic, globose, smooth, and 19.0–26.0 (mean 22.2) µm in diameter, with one per oogonium. The thickness of the oospore wall was 0.2–2.0 (mean 1.1) µm. The minimum, optimum, and maximum temperatures for growth on PCA were 0 °C, 22 °C, and 25 °C, with daily growth rates at 1.0 mm, 6.3 mm, and 5.7 mm, respectively (Figure 4). *Globisporangium* sp. 5 was phylogenetically closely related to *G. kandovanense, G. rostratifingens*, and *G. rostratum*, but was distinguished from these three related species by the size of its oogonia and the positions of its antheridium. *Globisporangium* sp. 5 was also distinguished from *G. rostratifingens* and *G. rostratum* by growing at 0 °C.

*Globisporangium* sp. 6 strain 10G26N1: Main hyphae were up to 5 µm in diameter. Sporangia were not observed. Hyphal swellings were observed in single culture (Figure 3f). Sexual reproductive organs and sporangia were formed neither in single nor dual culture. The minimum, optimum, and maximum temperatures for growth on PCA were 0 °C, 22 °C, and 28 °C, with daily growth rates at 0.9 mm, 7.1 mm, and 5.0 mm, respectively (Figure 4). The growth rate at 25 °C was 7.0 mm per day. Like *Globisporangium* sp. 5, sp. 6 is phylogenetically closely related with *G. kandovanense, G. rostratifingens*, and *G. rostratum*. Although the species identity was unclear for *Globisporangium* sp. 6, this species could be distinguished from *G. rostratifingens* and *G. rostratum* by growing at 0 °C. The species also differed from *G. kandovanense* by not forming sporangia.

All the *Globisporangium* strains obtained were identified as one of six species, i.e., *Globisporangium* sp. 1, ibid sp. 2 (=*G. polare*), ibid sp. 3, ibid sp. 4, ibid sp. 5, and ibid sp. 6, except for the strain 12G14W1. Strains of *Globisporangium* spp. 1 to 6 grew at 0 °C on agar plates and infected the *Sanionia* moss at 4 to 10 °C. Among the six species, only *Globisporangium* sp. 2 was a known species and was *G. polare* [22]. The other five remained unknown species. *G. polare* was first described from *Sanionia* moss with brown discoloration under snow cover in Longyearbyen, Spitsbergen Is., and has been found only in polar regions [22]. The phylogenetic position of the strain 12G14W1 was closest to *G. kandovanense* which was isolated from *Lolium perenne* with snow rot symptoms in a natural grassland in East Azerbaijan province, Iran [41]. The present results, together with previous reports, suggest that *Globisporangium* in *Sanionia* moss colonies in Ny-Ålesund not only has a unique species composition, but also shows adaptation to cold environments. Further study is needed to describe the new species for the unknown *Globisporangium* spp.

### 3.2. Infectivity to Sanionia Moss

**Figure 5:**
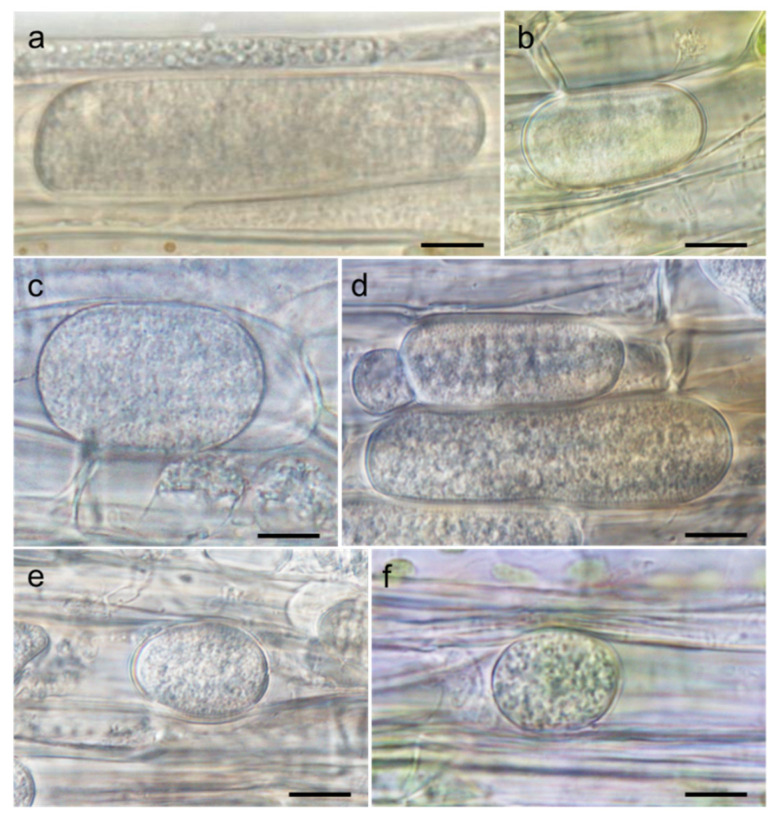
Production of sporangia and hyphal swellings of *Globisporangium* spp. isolated from a single colony of *Sanionia* moss in Ny-Ålesund, Spitsbergen Is., Norway in host plant tissues under an in vitro inoculation at 4 to 10 °C: (**a**) hyphal swelling of *Globisporangium* sp. 1 strain 10G16V2; (**b**) sporangium of *Globisporangium* sp. 2 (=*G. polare*) strain 18G12N1; (**c**) sporangium of *Globisporangium* sp. 3 strain 18C32N1; (**d**) sporangium of *Globisporangium* sp. 4 strain 18C32N1; (**e**) sporangium of *Globisporangium* sp. 5 strain 18G13N1; and (**f**) hyphal swelling of *Globisporangium* sp. 6 strain 18C14N1. Bars = 10 μm.

*Globisporangium* spp. 1, 2, 3, 4, and 6 infected the moss cells by penetration and colonization of mycelia at 4 °C and/or 10 °C (Table 1). Only one of the three strains tested of *Globisporangium* sp. 1 managed to colonize the moss cells, because the other two strains were lost when the test was done. Among the six species, *Globisporangium* spp. 1–4 consistently formed hyphae, oospores, and sporangia into the stem leaves of the moss cells (Table 1). At least one strain of all six groups produced sporangia or hyphal swellings inside the moss cells (Figure 5). All the strains infected the moss without showing any symptoms such as blight or discoloration of shoots and leaves until about 2 months after inoculation. The *Globisporangium* spp. were reisolated from the nonsymptomatic moss (Table 1).

Lévesque and de Cock [42] characterized phylogenetic clades of *Pythium* involving *Globisporangium*. Based on their clades, the *Globisporangium* spp. found in this study belong to clades E, F, and G [42]. *Globisporangium* sp. 1 belonged to clade F. This clade includes important crop pathogens such as *G. spinosum*, *G. irregulare*, *G. sylvaticum*, and *G. debaryanum*. *Globisporangium* spp. 2 (=*G. polare*), 3, and 4 belonged to clade G. This clade also includes important plant pathogens such as *G. iwayamai*, *G. paddicum*, and *G. okanoganense*, which cause snow rot of wheat and barley in Asia and the USA [25]. *Globisporangium* spp. 5 and 6 belonged to clade E, which includes weak pathogens of many plants [37]. This suggests that *Globisporangium* spp. 1 to 6 could be potential crop pathogens.

### 3.3. Isolation Pattern

**Figure 6:**
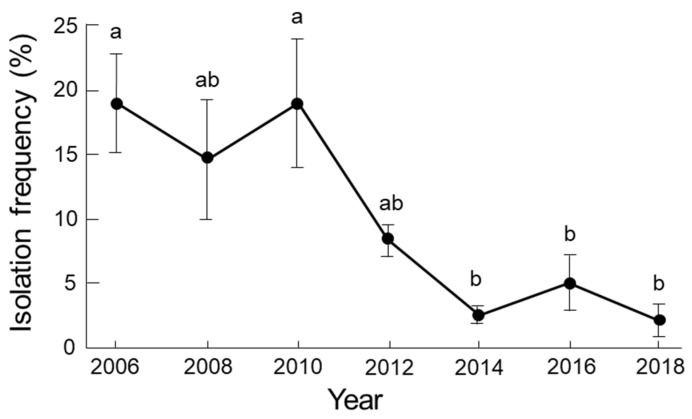
Changes of isolation frequency for the total population of *Globisporangium* spp. from a single colony of *Sanionia* moss in Ny-Ålesund, Spitsbergen Is., Norway from 2006 to 2018. The investigations were conducted in August each year. Isolation frequency was calculated as the number of moss shoots with isolated *Globisporangium* spp. divided by the total number of moss shoots examined. The average values with SE (*N* = 6) were shown. Values followed by the same letter are not significantly different according to Tukey’s HSD test (*p* < 0.05).

**Figure 7:**
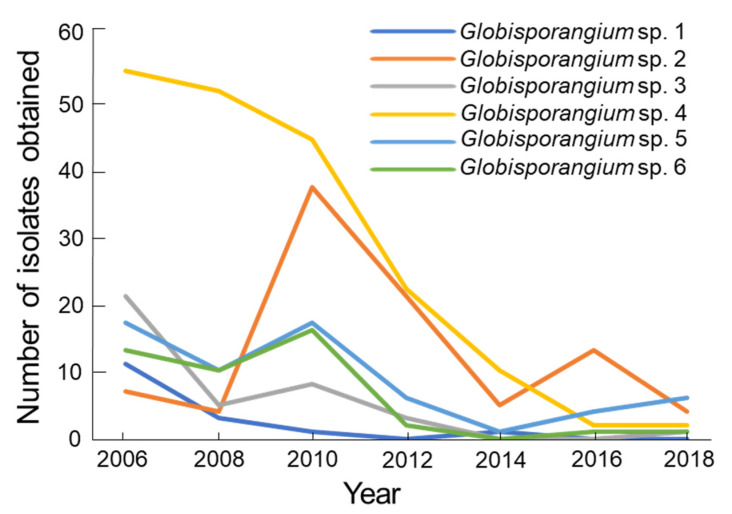
Total number of isolates of *Globisporangium* sp. 1, sp. 2 (=*G. polare*), sp. 3, sp. 4, sp. 5 and sp. 6 from a single colony of *Sanionia* moss at the north-side cliff of Ny-Ålesund, Spitsbergen Is., Norway from 2006 to 2018. The investigations were carried out in August each year for the six sampling plots shown in Figure 1.

Isolation frequency of the total population of *Globisporangium* spp. was maintained between 2006 and 2010, and significantly (*p* < 0.05) decreased from 2012 to 2018 (Figure 6). The total population was lowest in 2018 during the twelve-year period. The changes in the isolation pattern were different for the six *Globisporangium* spp. (Figure 7). *Globisporangium* spp. 1, 3, 4, and 6 consistently decreased from 2012 on. *Globisporangium* sp. 1 was not recorded in 2012, 2016, and 2018. *Globisporangium* spp. 2 (=*G. polare*) and 5 maintained their population, although the population differed from year to year.

Quantitative isolation from 2006 to 2018 demonstrated that total population of *Globisporangium* significantly decreased during the twelve-year period. Most of the *Globisporangium* spp. decreased their population. Only *Globisporangium* spp. 2 (=*G. polare*) and 5 showed little decreasing. The reason for the population decreasing is difficult to explain, but it may be influenced by climate changes in Arctic regions [43,44]. The influence of climate changes has already been recognized in the species composition and distribution of the Arctic vegetation [45,46]. *Globisporangium* spp. inhabiting Arctic regions are cold-adapted mesophiles rather than true psychrophiles (cold-loving), because they can grow at 20–25 °C. Mycelia of *Globisporangium* spp. are less freeze-resistant than those of fungi, even though a few isolates of *G. polare* are tolerant [28]. However, *Globisporangium* spp. can be highly tolerant to freezing when they have infected plant tissues [28]. The present in vitro study confirmed consistent infection of wet living moss by all six *Globisporangium* spp. under cold conditions. Previous and current results suggest that *Globisporangium* spp. found in the study site mainly increase their population during the summer period by infecting *Sanionia* moss, although they can grow at 0 °C under snow cover. Since *Globisporangium* requires wet conditions to produce hyphae, sporangia, and oospores [37], a consistent moist condition during the summer period is necessary to maintain its population. Romero et al. [3] reported that humidity is a primary driving factor for outbreaks of plant diseases caused by fungi and oomycetes. The recent continuous warming in the Arctic regions will decrease the diversity of mosses [11,46], which can be host plants of *Globisporangium* in the region. Better understanding of taxonomic and ecological features of the Arctic *Globisporangium* is needed, because they have unique species constructions and are probably vulnerable to climate changes.

## 4. Conclusions

At least six species of *Globisporangium* were found in single colony of the *Sanionia* moss in Ny-Ålesund, Spitsbergen Is., Norway. Among them, *G. polare* was the only known species, which has only been found in polar regions. The other five were unknown species and remain to be described as new species. All six species grew at 0 °C on an agar plate. All of them infected *Sanionia* moss under an in vitro inoculation test. Quantitative isolations of *Globisporangium* spp. from 2006 to 2018 showed that most of the species reduced their population over the recent decade at the study site. Much like other plant-parasitic oomycetes, the present *Globisporangium* spp. require a consistent moist condition to maintain their population. Recent climate change is influencing humidity in the Arctic region and could become a factor in the population reduction of the *Globisporangium* spp. Considering the unique species construction of *Globisporangium* found in this study, further evaluations are needed to provide better understanding of the taxonomic and ecological features of these species.

## Figures and Tables

**Table 1:** Infectivity of *Globisporangium* spp. from the *Sanionia* moss in Ny-Ålesund to *Sanionia uncinata* in an in vitro inoculation test.

Taxonomic Group	Strain	Temperature	Infection into the Host Plant Cells with;		Recovery from the Host Plant
		(°C)	Hyphae	Oospores or Sporangia	
*Globisporangium* sp. 1	10G16V2	4	+	++	+
*Globisporangium* sp. 2 (*G. polare*)	10G15W2	4	+	+	+
	18G12N1	10	+	+	+
*Globisporangium* sp. 3	10C17N1	4	+	++	+
	18C32N1	10	+	+	+
*Globisporangium* sp. 4	10G34N1	4	+	+	+
	18C32N1	10	+	+	+
*Globisporangium* sp. 5	10C12N1	4	−	−	−
	18G13N1	10	+	+	+
*Globisporangium* sp. 6	10G26N1	4	+	−	+
	18C14N1	10	+	+	+
Uninoculated		4	−	−	−
		10	−	−	−

++: Infection was found more than 50% of the plant part examined, +: infection was found less than 50% of the plant part examined, −: no infection. Recovery of *Globisporangium* spp. from stem-leaves was calculated from the number of stem leaves from which *Globisporangium* was recovered after 4 weeks of incubation at 4 °C.

## Data Availability

The data presented in present paper are available in this article.

## Supplementary Materials

The following are available online at https://www.mdpi.com/article/10.3390/microorganisms9091912/s1, Table S1: Information for *Globisporangium* strains used in the phylogenetic tree of Figure 2.
