# Supplementary material for: Identification and Isolation Pattern of Globisporangium spp. from a Sanionia Moss Colony in Ny-Ålesund, Spitsbergen Is., Norway from 2006 to 2018
Source: Microorganisms. 2021 Sep 9;9(9):1912. doi: 10.3390/microorganisms9091912 (PMC8467116; doi:10.3390/microorganisms9091912)
Supplement: Supplementary file 1 [file microorganisms-09-01912-s001.zip › Table S1 microorganisms-1345415_revised_2021_09_09.pdf]

Table S1. Information for *Globisporangium* strains used in the phylogenetic tree of Figure 2.

Species or taxonomic group	Strain	Time isolated	Location	ITS sequences		Reference
				Maximum identity with known species (%)	GenBank accession	
Globisporangium sp. 1	OPU1276	Jul. 2003	Ny-Ålesund, Norway	89.8	LC649254	Present study
Globisporangium sp. 1	8G11W1	Aug. 2008	Ny-Ålesund, Norway	89.8	LC644963	Present study
Globisporangium sp. 1	10G16V2	Aug. 2010	Ny-Ålesund, Norway	89.8	LC622526	Present study
Globisporangium sp. 2 (*G. polare*)	10G15W2	Aug. 2010	Ny-Ålesund, Norway	100	LC644964	Present study
Globisporangium sp. 2 (*G. polare*)	12G36V1	Aug. 2012	Ny-Ålesund, Norway	100	LC644721	Present study
Globisporangium sp. 2 (*G. polare*)	18G11V1	Aug. 2018	Ny-Ålesund, Norway	100	LC603672	Present study
Globisporangium sp. 2 (*G. polare*)	18G12N1	Aug. 2018	Ny-Ålesund, Norway	100	LC603666	Present study
Globisporangium sp. 2 (*G. polare*)	18G12N2	Aug. 2018	Ny-Ålesund, Norway	99.8	LC603667	Present study
Globisporangium sp. 3	10C17N1	Aug. 2010	Ny-Ålesund, Norway	93.0	LC644965	Present study
Globisporangium sp. 3	12G27W1-	Aug. 2012	Ny-Ålesund, Norway	93.0	12G27W1	Present study
Globisporangium sp. 3	18C29N1	Aug. 2018	Ny-Ålesund, Norway	93.3	LC603664	Present study
Globisporangium sp. 4	10G34N1	Aug. 2010	Ny-Ålesund, Norway	99.0	LC644966	Present study
Globisporangium sp. 4	12G28N1	Aug. 2012	Ny-Ålesund, Norway	99.0	12G28N1	Present study
Globisporangium sp. 4	18C32N1	Aug. 2018	Ny-Ålesund, Norway	99.1	LC603665	Present study
Globisporangium sp. 4	18G32N1	Aug. 2018	Ny-Ålesund, Norway	99.3	LC603671	Present study
Globisporangium sp. 5	10C12N1	Aug. 2010	Ny-Ålesund, Norway	96.0	LC644967	Present study
Globisporangium sp. 5	18G13N1	Aug. 2018	Ny-Ålesund, Norway	95.0	LC603668	Present study
Globisporangium sp. 5	18G15N1	Aug. 2018	Ny-Ålesund, Norway	96.7	LC603669	Present study
Globisporangium sp. 5	18G31N1	Aug. 2018	Ny-Ålesund, Norway	96.6	LC603670	Present study
Globisporangium sp. 5	18C17N2	Aug. 2018	Ny-Ålesund, Norway	96.1	LC603662	Present study
Globisporangium sp. 5	18C26N1	Aug. 2018	Ny-Ålesund, Norway	96.6	LC603663	Present study

Globisporangium sp. 5	18G37V1	Aug. 2018	Ny-Ålesund, Norway	95.5	LC603674	Present study
Globisporangium sp. 6	10G26N1	Aug. 2010	Ny-Ålesund, Norway	87.5	LC644968	Present study
Globisporangium sp. 6	12C19V2	Aug. 2012	Ny-Ålesund, Norway	87.5	LC644725	Present study
Globisporangium sp. 6	18C14N1	Aug. 2018	Ny-Ålesund, Norway	86.7	LC603661	Present study
Globisporangium sp.	12G14W1	Aug. 2012	Ny-Ålesund, Norway	98.6	LC644724	Present study
Globisporangium spinosum	CBS275.67	1967	Baarn, Netherlands		HQ643793	[40]
Globisporangium mamillatum	CBS251.28	1928	Netherlands		HQ643687	[40]
Globisporangium debaryanum	CBS752.96	Un-known	UK		HQ643519	[40]
Globisporangium terrestris	CBS112352	Un-known	Lille, France		HQ643857	[40]
Globisporangium sylvaticum	CBS453.67	1967	Georgia, USA		AY598645	[40]
Globisporangium polare	CBS118203	1999	Longyearbyen, Norway		KJ716859	[22,39]
Globisporangium canariense	CBS112353	Un-known	Canary Islands, Spain		HQ643482	[40]
Globisporangium iwayamai	CBS156.64	1962	Bundaleer North, Australia		HQ643669	[40]
Globisporangium violae	CBS159.64	1962	Bundaleer North, Australia		AY598706	[40]
Globisporangium okanoganense	CBS315.81	1977	Oregon, USA		HQ643714	[40]
Globisporangium paddicum	CBS698.83	1982	Fukui Pref., Japan		HQ643728	[40]
Globisporangium nagaii	CBS779.96	Un-known	Unknown		AY598705	[40]
Globisporangium middletonii	CBS528.74	1969	Zuidelijk Flevoland, Netherlands		HQ643694	[40]
Globisporangium multisporum	CBS470.50	1947	Illinois, USA		HQ643700	[40]
Globisporangium takayamanum	CBS122492	2004	Takayama, Japan		HQ643853	[40]
Globisporangium minus	CBS226.88	1985	Berkshire, UK		HQ643696	[40]
Globisporangium echinulatum	CBS281.64	1962	Caroline, Australia		HQ643531	[40]
Globisporangium rostratum	CBS533.74	1971	Oostelijk Flevoland, Netherlands		HQ643767	[40]
Globisporangium rostratifingens	CBS383.34	1933	Netherlands		HQ643762	[40]

Globisporangium kandovanense	CBS139567	Apr. 2014	Kandovan, Iran		KP938427	[41]
Pythium aphanidermatum	CBS28779	1979	Bulgaria		HQ643439	[40]
